# The Elusive Diagnosis: A Case of Extranodal Natural Killer (NK)/T-Cell Lymphoma

**DOI:** 10.7759/cureus.104482

**Published:** 2026-03-01

**Authors:** Amanda Mock, Sebastián Sepúlveda, Julio Viquez, Abdiel Bracho, Elizabeth D Orcy

**Affiliations:** 1 Department of Otolaryngology, Complejo Hospitalario Metropolitano Dr. Arnulfo Arias Madrid, Caja de Seguro Social, Panama City, PAN; 2 Faculty of Medicine, Universidad de Panama, Panama City, PAN; 3 Department of Pathology, Complejo Hospitalario Metropolitano Dr. Arnulfo Arias Madrid, Caja de Seguro Social, Panama City, PAN

**Keywords:** biopsy, case report, epstein-barr virus infection, extranodal nk/t-cell, lymphoma, nose neoplasms

## Abstract

Extranodal natural killer (NK)/T-cell lymphoma is a rare malignant tumor, more prevalent in Asia and Latin America, and associated with Epstein-Barr virus infection. Its diagnosis is challenging due to its clinical presentation, which may mimic chronic sinusitis, dacryocystitis, or granulomatous diseases. This lymphoma follows a rapid and aggressive course, with a poor prognosis. We report the case of a 71-year-old man presenting with nasal obstruction, clear rhinorrhea, and facial heaviness. Initially, acute dacryocystitis and periorbital cellulitis were suspected and treated with antibiotics; however, the patient showed a poor response. After multiple consultations and unsuccessful treatments, a biopsy confirmed the diagnosis of extranodal NK/T-cell lymphoma of the nasal type. Histopathological and immunohistochemical analysis revealed an infiltrate of atypical lymphocytes positive for cluster of differentiation 2 (CD2), cluster of differentiation 56 (CD56), T-cell intracellular antigen-1 (TIA-1), and Epstein-Barr virus-encoded RNA (EBER), with prominent tissue necrosis. This case highlights the diagnostic challenges and aggressive clinical course of sinonasal NK/T-cell lymphoma. Its initial presentation can mimic common inflammatory conditions, requiring a high index of suspicion. Standard treatment includes combined chemotherapy and radiotherapy, yet the prognosis remains poor. This report aims to raise awareness of this entity and emphasize the importance of considering it in atypical or treatment-refractory cases of orbital cellulitis, bacterial sinusitis, and other inflammatory conditions.

## Introduction

Extranodal natural killer (NK)/T-cell lymphoma, nasal type, is classified within mature T- and NK-cell and predominantly affects males in their fifth or sixth decade of life [1,2]. Its etiology is closely related to infection by the Epstein-Barr virus, with universal positivity for Epstein-Barr virus-encoded RNA (EBER) found in the tumor cells [3].

Its initial presentation can be indistinguishable from common inflammatory pathologies, such as chronic sinusitis, dacryocystitis, periorbital cellulitis, or persistent nasal obstruction [2,4]. A sluggish evolution, poor response to antibiotic treatment, and the appearance of necrosis or ulcerated lesions should alert the specialist to the possibility of an underlying malignant etiology. It is not uncommon for the definitive diagnosis to be delayed, sometimes after multiple consultations or failed treatments [2]. The histopathological and immunohistochemical study reveals an angiocentric infiltrate of atypical lymphocytes that are cluster of differentiation 2 positive (CD2+), cluster of differentiation 56 positive (CD56+), T-cell intracellular antigen-1 positive (TIA-1+), with EBER positivity and prominent tissue necrosis [1,3,4]. Staging requires imaging studies, such as fluorodeoxyglucose positron emission tomography/computed tomography (FDG PET-CT) [4]. Standard treatment for the disease consists of chemoradiotherapy [4]. In this context, we present the case of a 71-year-old male patient with initial suspicion of acute left dacryocystitis and left periorbital cellulitis with partial response to conventional treatment, where the diagnosis of extranodal NK/T-cell lymphoma, nasal type, was confirmed by a biopsy.

## Case presentation

A 71-year-old male presented with a two-month history of obstruction in the left nostril, clear rhinorrhea, and ipsilateral facial heaviness and two weeks of painful swelling of the left lower eyelid.

**Figure 1:**
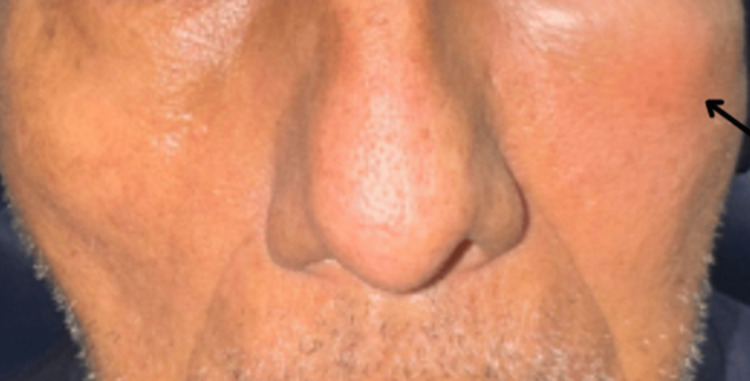
Onset physical examination Left lower eyelid and maxillary edema measuring 6 x 3 cm.

**Figure 2:**
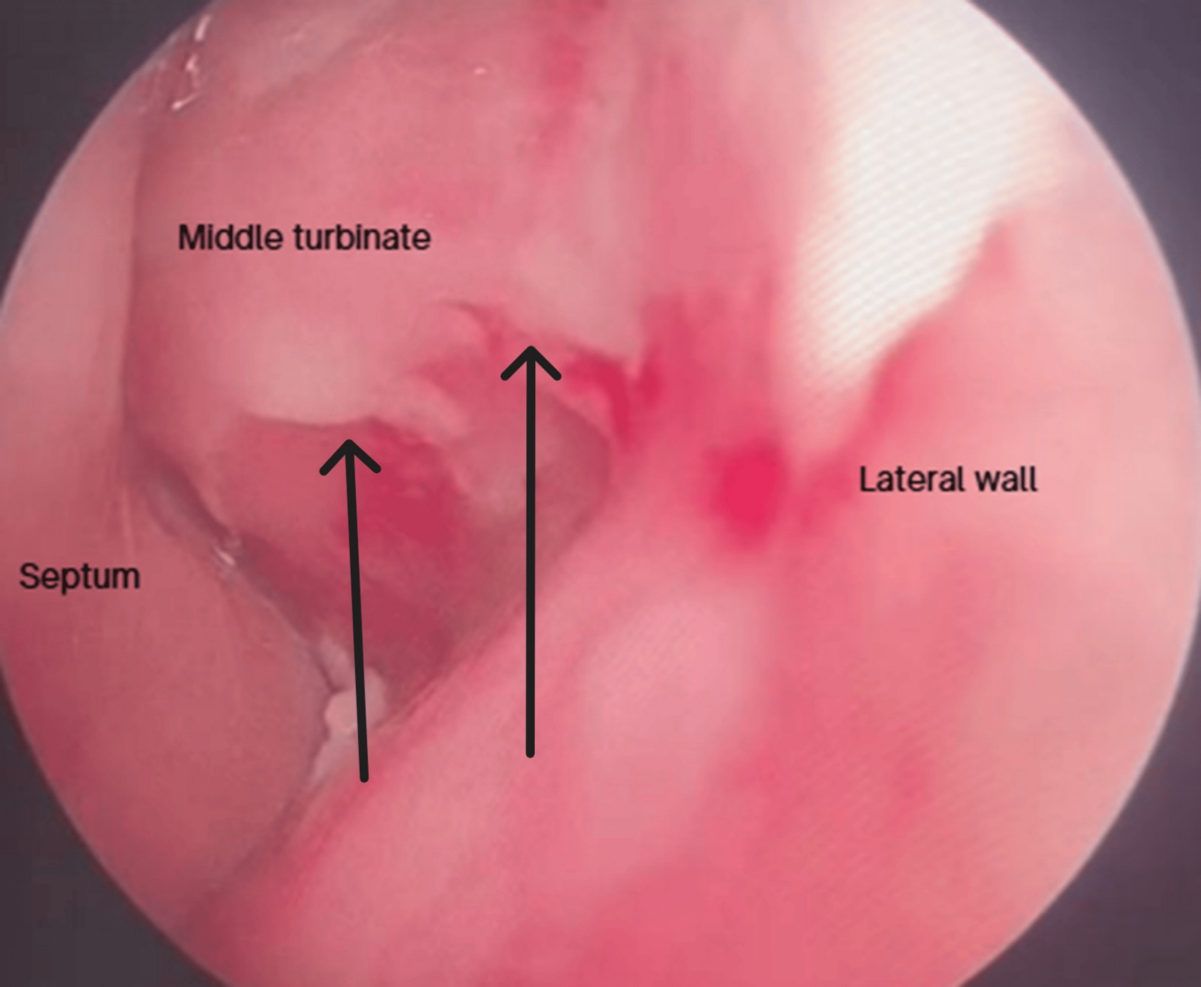
Onset nasal endoscopy White discharge on the lateral wall and the middle turbinate.

**Figure 3:**
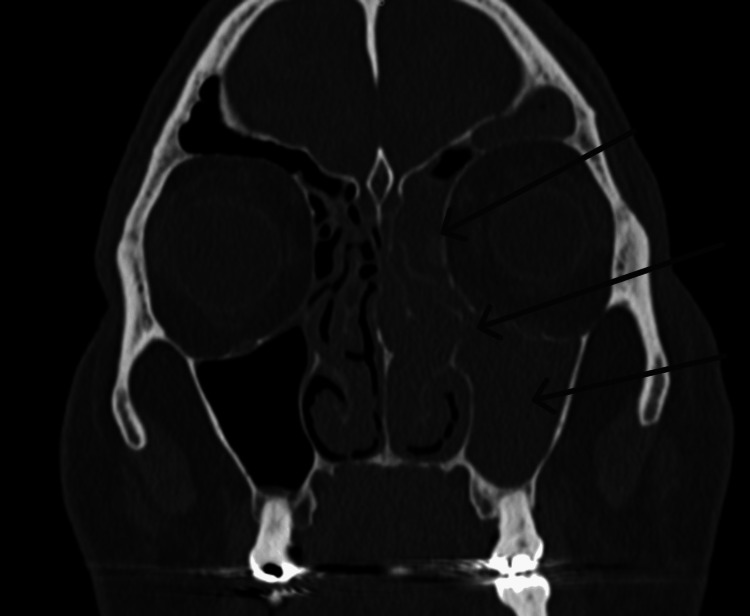
Onset coronal sinus CT scan Evidence of chronic inflammatory changes in the medial wall of the maxillary sinus at the level of the middle turbinate and tissue density in the left frontoethmoidomaxillary region.

Upon physical examination, he presented with edema and slight erythema of the left lower eyelid and maxilla, measuring 6 × 3 cm (Figure 1). Endoscopically, thickened mucosa was evident over the medial part of the left inferior and middle turbinate (Figure 2). The tomography showed bone thinning of the middle nasal turbinate and part of the medial wall of the left maxillary sinus, tissue density in the left frontoethmoidomaxillary region, and inflammatory changes in the inferomedial extraconal fat adjacent to the lacrimal canaliculi, which extended to the soft tissues of the infraorbital and periorbital region, with skin thickening up to 0.8 cm (Figure 3). He was managed with intravenous (IV) levofloxacin and metronidazole, with improvement of the lower eyelid edema and nasal obstruction, leading to discharge.

**Figure 4:**
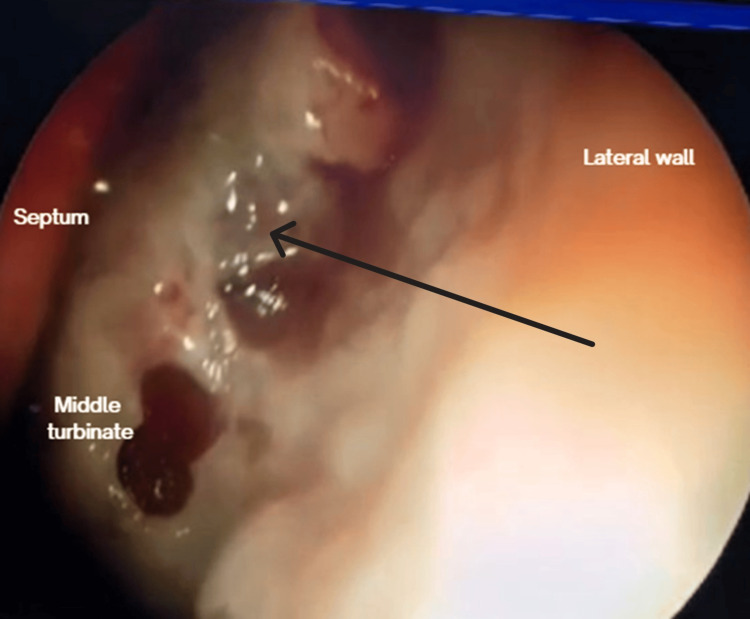
Follow-up nasal endoscopy 15 days after discharge Friable mucosa with necrotic discharge

**Figure 5:**
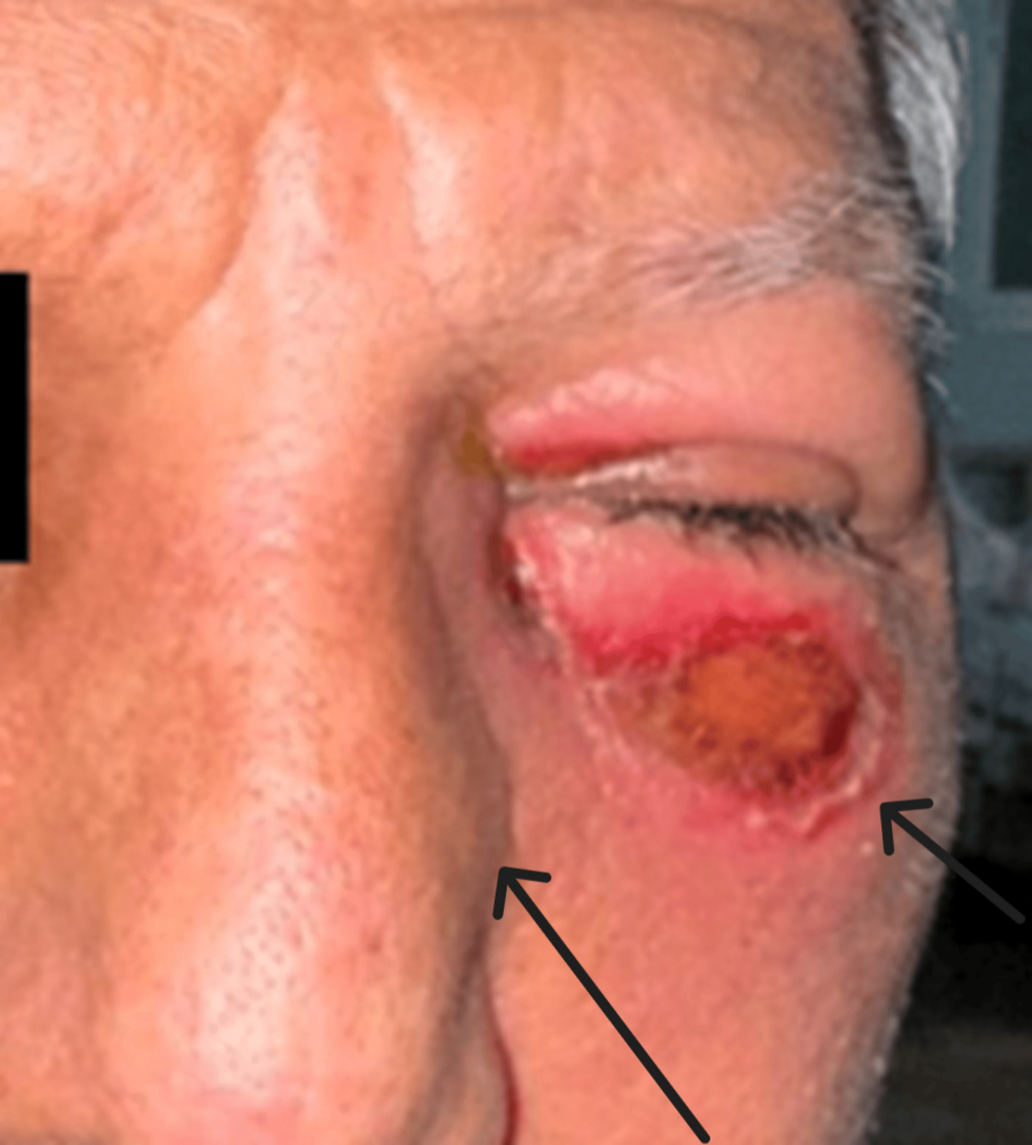
Skin ulceration 21 days after discharge Ulceration of the left malar skin with indurated borders close, located proximal to the inferior eyelid.

**Figure 6:**
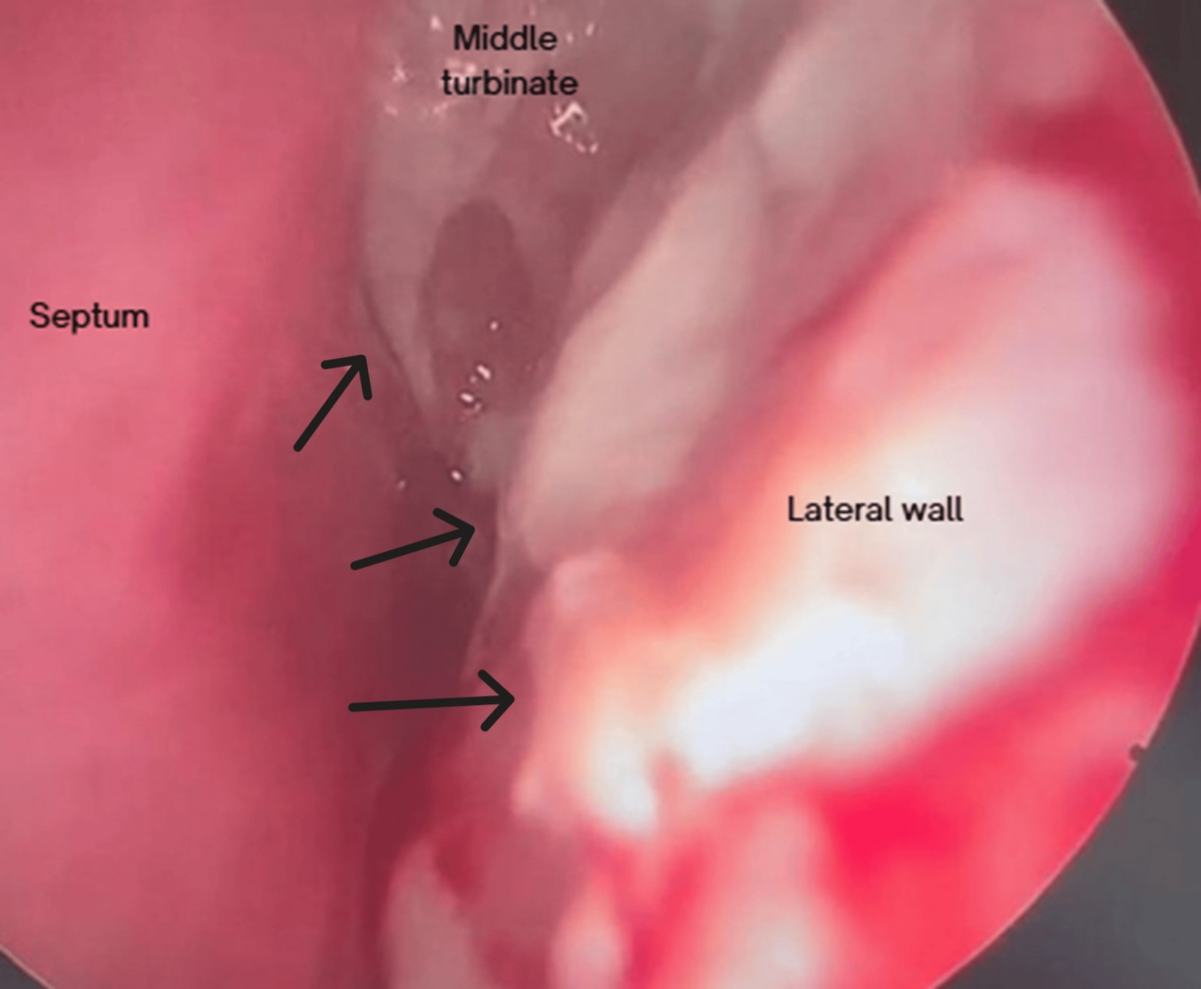
Endoscopy with granulation tissue 21 days after discharge

**Figure 7:**
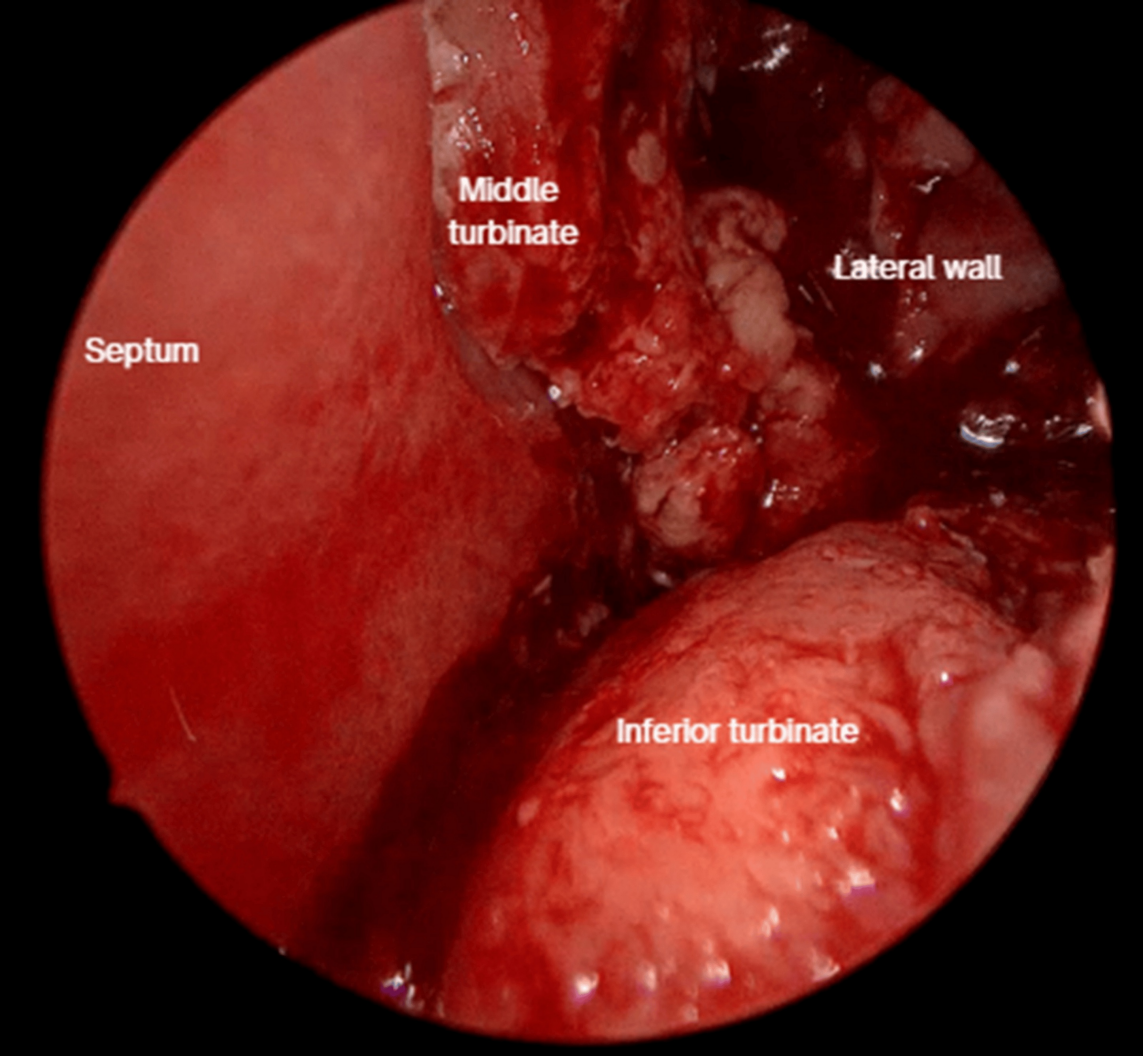
Functional endoscopic sinus surgery + dacryocystectomy + biopsy

**Figure 8:**
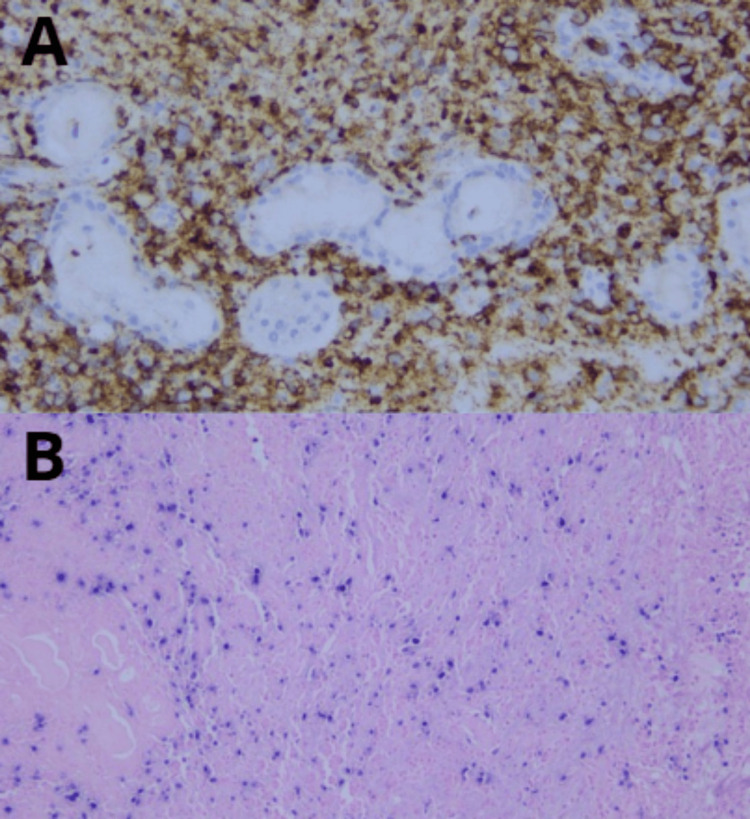
(A) Cluster of differentiation 2 positive (CD2+). (B) Epstein-Barr virus-encoded RNA positive (EBER+)

After 15 days, on follow-up, the patient presented friable and thickening of the mucosa on the left inferior and middle turbinate and left anterior lateral nasal wall (Figure 4). There was also a recurrence of left lower eyelid edema within seven days, associated with clear rhinorrhea, hyposmia, left hemifacial heaviness, and ipsilateral epiphora following a respiratory infection. The lower eyelid edema limited eye opening and extended to the left cheekbone (Figure 5). A nasal endoscopy one month after onset exposed persistent granulation tissue and fibrin over the inferior turbinate, lateral wall, and middle turbinate (Figure 6); new CT imaging reported persistent tissue density in the left frontoethmoidomaxillary region, with contrast enhancement and thickening of the left lacrimal duct, edema of infraorbital soft tissues, and malar skin. The patient was readmitted and started on IV vancomycin. During treatment, the patient developed an ulcerated lesion on the left malar region with indurated borders and progressive worsening of left palpebral edema. Nasal discharge cultures were taken and found positive for *Candida albicans*. He underwent functional endoscopic sinus surgery + dacryocystectomy + biopsy of the left nasal mucosa (Figure 7). The biopsy reported extranodal NK/T-cell lymphoma, nasal type, with expression of CD2 (Figure 8A), CD56, TIA-1, and granzyme B, with positive in situ hybridization for EBER, and abundant necrotic tissue (Figure 8B). Management by hematology was initiated, but the patient died before completing treatment two months after the initial onset.

## Discussion

This case highlights the diagnostic difficulties and the aggressive and elusive clinical course of extranodal NK/T-cell lymphoma, nasal type, since its initial presentation mimics acute dacryocystitis, periorbital cellulitis, and chronic sinusitis, as well as necrotizing sinonasal pathologies, such as necrotizing fungal rhinosinusitis, leishmaniasis, and other types of cancer.

A high index of suspicion is required for atypical or treatment-refractory presentations. Frequent symptoms consist of nasal obstruction (70%), epistaxis (47%), and B symptoms (31-52%) [5]. The most involved tissue is the nasal cavity (>97%), and it can present on the facial skin (15%) [5]. Advanced nasal T-cell lymphoma has been found to be associated with septal destruction and perforation [5].

Despite initial improvement of lower eyelid edema due to the IV levofloxacin and metronidazole therapy, our patient presented with a facial skin ulcer that progressed and a recurrence of nasal symptoms. He presented dermatological manifestations described in the literature as an ulcer with indurated borders inferior to the left lower eyelid [6,7]. The course involved the evolution of progressive nasal inflammation to necrosis, as described in the literature. His tomographic findings were nonspecific [5,6].

Extranodal NK/T cell-lymphoma presents as a mass on nasal endoscopy, associated with erosion, crusting, friability upon palpation, and granulation. Obtaining a deep biopsy is crucial for distinguishing it from other causes of pathology that affect the mucosa more superficially [6]. The diagnosis was made by histopathological and immunohistochemical identification of CD2+, CD56+, TIA-1+, and EBER+. Pathology of the extranodal T/NK-cell lymphoma, nasal type, is normally presented with expression of CD3ε (polyclonal) in the infiltrating cells; with partial expression of CD2, CD5, CD7, and, to a lesser extent, CD8 and CD56; with a proliferation index of 70% according to Ki-67; and with positive in situ hybridization for EBER [8]. It has been shown that biopsies under general anesthesia, resection of the inferior turbinate, and performing a Caldwell-Luc procedure are superior to Tru-Cut biopsy [6].

## Conclusions

This case report underscores the critical challenge in the timely diagnosis of extranodal NK/T-cell lymphoma, nasal type, particularly in elderly patients presenting with seemingly common ophthalmological and sinonasal inflammatory conditions. The patient's initial presentation of acute dacryocystitis, periorbital cellulitis, and chronic sinusitis mimicked benign or fungal pathologies, leading to a significant diagnostic delay. This case serves as a crucial reminder to specialists to consider this rare but devastating etiology and proceed with biopsy when common sinonasal or periorbital inflammatory conditions fail to resolve completely.
